# Simultaneous PCR detection of *Paenibacillus larvae* targeting insertion sequence IS256 and *Melissococcus plutonius* targeting pMP1 plasmid from hive specimens

**DOI:** 10.1007/s12223-023-01125-0

**Published:** 2024-01-05

**Authors:** Katerina Vlkova, Tomas Erban, Martin Kamler, Dalibor Titera, Ibrahim Bitar, Jaroslav Hrabak

**Affiliations:** 1 Biomedical Center, Faculty of Medicine in Pilsen, Charles University, alej Svobody 76, 323 00 Pilsen, Czech Republic; 2 Department of Microbiology, Faculty of Medicine in Pilsen, Charles University, alej Svobody 80, 323 00 Pilsen, Czech Republic; 3 Crop Research Institute, Prague, Czech Republic; 4 Bee Research Institute at Dol, Libcice nad Vltavou, Czech Republic

**Keywords:** *Paenibacillus larvae*, *Melissococcus plutonius*, *Apis mellifera*, Multiplex PCR, American foulbrood, European foulbrood

## Abstract

*Paenibacillus larvae* and *Melissococcus plutonius* represent the most threatening bacterial diseases of honeybee (*Apis mellifera*)—American and European foulbrood, respectively. For efficient control of those diseases, rapid and accurate detection of the pathogens is crucial. Therefore, we developed a novel multiplex PCR method simultaneously detecting both pathogens. To design and optimize multiplex PCR reaction, four strains of *P. larvae* representing four ERIC genotypes I–IV (strain DSM 7030—ERIC I, DSM 25430—ERIC II, LMG 16252—ERIC III, DSM 3615—ERIC IV) were selected. Those strains were fully sequenced using long-read sequencing (Sequel I, Pacific Biosciences). For *P. larvae*, the multicopy insertion sequence IS*256* identified in all genotypes of *P. larvae* was selected to provide high sensitivity. *M. plutonius* was detected by plasmid pMP1 sequence and the virulence verified by following detection of ETX/MTX2 toxin responsible for pore formation in the cell membrane. As an internal control, a gene encoding for major royal jelly protein 1 specific for honeybees was selected. The method was validated on 36 clinical specimens collected from the colonies suffering from American and European foulbrood in the Czech Republic. Based on the results, sensitivity of PCR was calculated to 93.75% and specificity to 100% for *P. larvae* diagnosed from hive debris and 100% sensitivity and specificity for honeybee workers and larval scales as well as for diseased brood infected by *M. plutonius*.

## Introduction

*Paenibacillus larvae* is a worldwide spreading pathogen of honeybee (*Apis mellifera*) causing American foulbrood (AFB) (Hansen and Brødsgaard 1999). Due to the high stability of the spores in the environment, it represents a disease with high epizootic potential (Forsgren et al. 2008; Genersch et al. 2006; Genersch 2010). In the colony, it infects only the bee brood, causing a complete disintegration of the larvae into a spore-rich ropy mass (Ebeling et al. 2016; Poppinga and Genersch 2015). The spores are mostly spread by infected honey among the colonies due to foraging, robbing, and beekeeper’s activity introducing contaminated material (e.g., frames, wax foundations, honey). As the spores of *P. larvae* are not virulent for adult bees (Lindström et al. 2008), they can be silently introduced into the colonies.

Another severe bacterial brood disease, European foulbrood (EFB), is caused by *Melissococcus plutonius*. Like *P. larvae*, *M. plutonius* is ingested along with contaminated food, followed by the injury of the peritrophic matrix and epithelial cells of the midgut. Contrary to *P. larvae*, *M. plutonius* does not form the spores and its stability in the environment is therefore affected. Despite that fact, significant mortality of infected colonies resulting in high economic impact has been also observed.

Therefore, rapid detection of *P. larvae* and *M. plutonius* is crucial for intervention to control American and European foulbrood, respectively. Traditional methods are based on the cultivation of those bacteria from hive debris, infected brood, bees, or honey (Reynaldi and Allipi 2006). Those methods require a few days to obtain results. Furthermore, for *M. plutonius*, an anaerobic atmosphere is required. Thus, diagnostics based on PCR can provide rapid results with high sensitivity and specificity. The first application of PCR for the detection of *P. larvae* was described by Govan et al. (1999), targeting a specific region of the 16S rRNA gene. Other research groups used the same target (16S rRNA gene) and optimized the method (Dobbelaere et al. 2001; Alippi et al. 2002; Piccini et al. 2002; Ryba et al. 2009). To increase sensitivity and specificity of molecular detection of *P. larvae*, other targets (KAT and iC9) as well as a nested format of PCR by two subsequent amplifications were proposed iC9 (Allipi et al. 2004; De Graaf et al. 2006; Han et al. 2008; Martínez et al. 2010; Chagas et al. 2010; Rossi et al. 2018; Lauro et al. 2003). For *M. plutonius*, PCR also represents an efficient tool for laboratory confirmation of European foulbrood (Arai et al. 2014; Garrido-Bailón et al. 2013; Biova et al. 2021, Sopko et al. 2019). Similar to *P. larvae*, PCR can be also focused on a specific sequence of the 16S rRNA gene (Garrido-Bailón et al. 2013). There are also reports describing the simultaneous multiplex detection of both pathogens, e.g., *P. larvae* and *M. plutonius* (Garrido-Bailón et al. 2013; Okamoto et al. 2022).

In this study, we used results from long-read whole-genome sequencing (Sequel I, Pacific Biosciences) and GenBank database to design a specific multiplex PCR detecting *P. larvae* and *M. plutonius*, pathogens causing American and European foulbrood, respectively. Comparing with previously published methods, we used other targets that enhance the sensitivity of the method, especially for the detection of *P. larvae*.

## Material and methods

### Bacterial strains and specimens

For development and optimalization of the PCR method, we selected four strains of *P. larvae* representing four ERIC genotypes I–IV (strain DSM 7030—ERIC I, DSM 25430—ERIC II, DSM 3615—ERIC IV obtained from Strains Collection of Leibniz Institute—Deutsche Sammlung von Mikroorganismen und Zellkulturen GmbH, Braunschweig, Germany, and LMG 16252—ERIC III obtained from Collection strains Culture Collection of the Laboratorium voor Microbiologie, Universiteit Gent, Belgium). For further confirmation of target specificity and sensitivity, a culture collection of 16 previously identified *P. larvae* isolates recovered from bee colonies in the Czech Republic was used.

For validation of the method, 25 blind samples of hive debris, 5 samples of bee workers (ca. 10 bees per sample), and 3 larval scales from colonies suspected of American foulbrood (colonies with clinical symptoms or colonies located on the same apiary with no visible symptoms) were selected. *M. plutonius* isolates as well as clinical specimens (diseased larvae) were obtained from three colonies suffering from European foulbrood recovered from the Czech Republic.

### Cultivation of *P. larvae* and *M. plutonius* and identification

For cultivation of *P. larvae*, PVX agar (bioMérieux, Prague, Czech Republic) was used and incubated in the atmosphere with 10% CO_2_ at 35 °C for 1–3 days until visible colonies were detected.

One gram of the hive debris was diluted by 10 mL of sterile distilled water and incubated at 90 °C for 6 min. After cooling to room temperature, 1 mL of toluene was added, and the mixture was vortexed for 2 min. Cultivation medium was inoculated by 100 μL of a water fraction. Similarly, 100 μL of the same fraction was used for three serial dilutions in physiological saline followed by inoculation of cultivation media to obtain quantitative results. Bee workers and larval scales were homogenized in physiological saline by a glass tissue homogenizer. One hundred microliters of the homogenate was inoculated and cultivated.

For cultivation of *M. plutonius*, Schaedler agar with 5% sheep blood (bioMérieux, Prague, Czech Republic) was cultivated in an anaerobic atmosphere standardly developed by gas generating sachet (Oxoid™ AnaeroGen™ 2.5 L Sachet, Thermo Fisher Scientific, MA, USA) until the colonies were visible (Forsgren et al. 2013).

Identification of suspected *P. larvae* and *M. plutonius* colonies was performed by a matrix-assisted laser desorption/ionization time-of-flight mass spectrometry (MALDI-TOF MS) using MicroFlex LT (Bruker Daltonik, Bremen, Germany) and MBT Compass software. As a matrix, alpha-cyano-4-hydroxycinnamic acid was used at a recommended concentration of 10 mg/mL (Bruker Daltonik, Bremen, Germany) in 50% acetonitrile (Sigma-Aldrich, Prague, Czech Republic) and trifluoroacetic acid (2.5%, v/v, Sigma-Aldrich, Prague, Czech Republic).

### DNA sequencing

DNA from fresh bacterial culture (colonies growing 48 h) was extracted according to the method described by Rossi et al. (2018). Sequel I platform (Pacific Biosciences, California, USA) was used for whole-genome sequencing. Library preparation was done following the microbial multiplexing protocol according to the manufacturer instructions for sheared DNA. Shearing was performed using g-TUBEs (Covaris, USA), and no size selection was done during the library preparations. Open reading frame (ORF) was predicted using RAST 2.0 (Brettin et al. 2015) with default parameters combined with BLASTP/BLASTN (Boutet et al. 2013), and insertion sequences were identified using the ISfinder database (http://www-is.biotoul.fr/). Comparative genome alignments were performed using Mauve (version 2.3.1).

### Primer design and real-time PCR

Primers for real-time PCR and the respective probes were designed by the Primer-BLAST (available at http://www.ncbi.nlm.nih.gov). As a target gene, IS*256* (WP_024095274.1) was selected due to its multiple presence in the genome of *P. larvae* (e.g., DSM7030 contains 84 copies, DSM25430 contains 229 copies). We also identified IS*256* in all sequences available in GenBank. To check the specificity, the target sequence as well as primer and probe sequences was analyzed in silico by Nucleotide BLAST (available at http://www.ncbi.nlm.nih.gov). After amplification, specificity of the amplified PCR product was confirmed by Sanger sequencing followed by the analysis using the same tool. Sensitivity was determined by a serial dilution from an initial concentration of *P. larvae* culture of genotype ERIC I DSM 7030. For *M. plutonius*, primers and probes were designed to target a specific sequence of the plasmid pMP1 (Nr. NC_015517). That sequence was found to be unique for *M. plutonius*, and we assumed its multicopy presence in the bacterial cell due to the location on a plasmid. To verify putative virulence of the strains, we proposed primers and probe target ETX/MTX2 family pore-forming toxin (Nr. NZ_CM003360.1). To control the PCR detection of *P. larvae* and *M. plutonius* including specimen collection from a beehive, DNA isolation and amplification, an internal control was designed. That internal control targets the major royal jelly protein 1 gene (*mrjp1*) that should be amplified in all specimens from beehives.

To prepare positive controls, PCR products representing all targets were cloned into the pTrcHis TOPO™ TA Expression Kit (Thermo Fisher Scientific, MA, USA) according to manufacturer recommendations.

Newly designed primers and probes are mentioned in Table 1. Multiplex PCR reaction was prepared using the TaqMan^®^ Multiplex Master Mix according to manufacturer recommendations (Thermo Fisher Scientific, MA, USA). PCR amplification was performed according to the following conditions: initial denaturation at 95 °C for 30 s, denaturation at 95 °C for 3 s, annealing with the measurement of fluorescence at 60 °C for 20 s repeated 40 times. The ETX/MTX2 family pore-forming toxin gene was amplified in a simplex PCR reaction, master mix and PCR conditions were identical.

**Table 1:** Sequences of PCR primers and probes

**Target organism/sequence**	**Primer**	**Sequence 5′ → 3′**	**Amplicon length**	**Target gene**	**Concentration in PCR reaction (μmol/L)**
**Multiplex PCR**					
*Paenibacillus larvae*	P_larvae_IS256_F	TCCACGTCCTTTTAGAGAGCTG	417 bp	*IS*256	0.250
	P.larvae_IS256_R	GACGGGAAGTTCACGACAG			0.250
	P_larvae_IS256_P	[FAM]-CGGAAAGAGGCTGTCTGCCAGG-[BHQ]			0.125
*Melissococcus plutonius*	M_plutonius_pMP1_F	TCCGACTATGAAGGGACGAAGAGA	413 bp	pMP1	0.250
	M_plutonius_pMP1_R	CGACATAAGCCATATCAAGCTGGG			0.250
	M_plutonius_pMP1_P	[JOE]-GGTCATTGCCAAGCTTTTATCTGC-[BHQ1]			0.125
Major royal jelly protein 1	MRJP1_F	CTCTTCTTCGGTTTGGTGGGCG	241 bp	mrjp1	0.150
	MRJP1_R	CAAAGACAAGCGTTGCATCGTATC			0.150
	MRJP1_P	[ROX]-GCTCTTGGCTGCTGGAACGA-[BHQ2]			0.125
**Simplex PCR**					
	MP_ETX_F	AGCAGATACCTATCAAGGGT	477 bp	ETX/MTX2	0.250
	MP_ETX_R	AAATTTTTACCGGTTGCAGT			0.250
	MP_ETX_P	[FAM]-ACCAGGGGAATTAAATTTGGTGG-[BHQ1]			0.125

### Nucleotide sequence accession numbers

The nucleotide sequences of the genomes and plasmids were deposited and are available in GenBank under the BioProject number PRJNA1006626.

## Results

Based on whole-genome sequencing and analysis of sequences available at GenBank (http://www.ncbi.nlm.nih.gov/GenBank), we identified possible targets for PCR identification. As a proper sequence to identify *P. larvae*, we selected insertion sequence IS256 located on the chromosome of *P. larvae* in multiple copies (84 copies in DSM7030, 229 copies in DSM25430). The use of a high copy number gene should increase the sensitivity of the detection method. The PCR was initially optimized and tested on four mentioned strains of *P. larvae* of ERIC representing four genotypes (I–IV) and *M. plutonius* (V1538) isolated from the Czech Republic in triplicates. Sensitivity of the detection of *P. larvae* was further tested using a sample DSM 7030 (ERIC I genotype). DNA of this strain was decimal diluted with an initial concentration of 60 ng/μL. The hypothesis of higher sensitivity using multicopy IS256 as a target comparing with the sequences present in a genome in a single copy was confirmed, allowing the detection of total *P. larvae* DNA concentration of 60 fg/μL (Table 2). Beyond excellent sensitivity, the assay demonstrated an excellent linearity. Similar sensitivity and linearity were observed for *M. plutonius* as well (Table 2).

**Table 2:** Results of qPCR of *P. larvae* DSM 7030 and *M. plutonius* in diluted samples

**DNA concentration**	***C***_**t**_***P. larvae***	**C**_**t**_***M. plutonius***
60 ng/μL	11.27	13.25
6 ng/μL	15.33	16.02
600 pg/μL	19.08	20.43
60 pg/μL	22.57	24.18
6 pg/μL	26.12	27.36
600 fg/μL	29.80	30.94
60 fg/μL	32.88	33.12
Negative control	Not detected	Not detected

Validation was performed on 25 samples of hive debris and larvae from three colonies suffering from American foulbrood (Table 3). The samples of hive debris were quantitatively cultivated. Among those samples, 9 were cultivation as well as PCR negative. In the sample Nr. D9, cultivation identified 1 × 10^4^ CFU/g but PCR of IS256 showed a negative result even though internal control was sufficiently amplified. The specimen, however, was significantly moldy, and thus, DNA extraction from *P. larvae* spores could be influenced by the inhomogeneity of the sample. The target sequences of the PCR range between 241 and 477 bp. As demonstrated in Table 2, PCR can detect *P. larvae* in specimens with very low quantity (1 × 10^2^ CFU/g); therefore, it was not necessary to modify primer sequences and to shorten the amplicon lengths. The *C*_*t*_ values ranged from 13 to 31 demonstrating good reliability with the spore concentration in the sample. In the specimen Nr. D17, the concentration of the spores determined by cultivation was 1 × 10^2^ CFU/g, but PCR positivity was demonstrated at *C*_*t*_ 29.49. The discrepancy may be from a lack of spore homogeneity in the debris.

**Table 3:** Comparison of results of cultivation and qPCR of *Paenibacillus larvae* recovered from 25 hive debris, 5 bee workers, and 3 larval scale samples

**Sample Nr**	**Cultivation (CFU/g)**	qPCR **(***C*_***t***_**)** ***P. larvae***	**qPCR** **(***C*_***t***_**)** ***mrjp1*** **(internal control)**
***Hive debris***			
D1	Negative	Negative	34.56
D2	1 × 10^2^	29.25	26.76
D3	1 × 10^4^	18.16	34.35
D4	Negative	Negative	27.81
D5	1 × 10^3^	24.96	28.17
D6	1 × 10^2^	31.47	27.66
D7	Negative	Negative	24.38
D8	1 × 10^5^	13.19	38.01
D9	1 × 10^4^	Negative	28.25
D10	1 × 10^4^	25.64	29.12
D11	1 × 10^3^	28.55	26.65
D12	Negative	Negative	28.72
D13	Negative	Negative	24.66
D14	1 × 10^5^	12.57	32.87
D15	1 × 10^3^	29.02	29.05
D16	1 × 10^2^	25.11	25.41
D17	1 × 10^5^	29.49	30.15
D18	1 × 10^4^	17.89	31.25
D19	1 × 10^3^	26.98	29.83
D20	Negative	Negative	26.52
D21	Negative	Negative	25.90
D22	1 × 10^2^	28.65	26.65
D23	Negative	Negative	24.35
D24	1 × 10^3^	29.26	27.06
D25	Negative	Negative	32.25
***Bees (workers)***			
B1	Positive	16.56	18.56
B2	Positive	24.31	12.38
B3	Negative	Negative	12.66
B4	Negative	Negative	14.34
B5	Negative	Negative	14.78
***Larval scale***			
S1	Positive	13.15	23.42
S2	Positive	12.28	36.08
S3	Positive	14.25	31.39

In all samples, including bee workers and larval scales, internal control (*mrjp1*) was successfully amplified (see Table 3). In all negative samples, the *C*_*t*_ was < 35. Therefore, the *C*_*t*_ threshold of the internal control amplification can be set up to 35.00. In the specimens with positive PCR amplification of *P. larvae* or *M. plutonius*–specific sequences, positive amplification of the internal control is not a mandatory condition to interpret the result as the validity of PCR can be indicated by a detection of the pathogen. Simultaneous amplification of internal control together with pathogen targets did not influence the sensitivity of multiplex PCR (increasing *C*_*t*_ by < 2.00).

Based on the results, sensitivity of PCR was calculated to 93.75% and specificity to 100% for *P. larvae* diagnosed from hive debris. All specimens of larvae infected by *M. plutonius* were identified as positive with a *C*_*t*_ value between 24 and 29.

## Discussion

To prevent the spread of American and European foulbrood in apiaries, it is crucial to perform active surveillance. Diagnosis is based on clinical symptoms of both diseases in the colonies followed by microbiological identification (cultivation, taxonomic identification) of the pathogens (de Graaf et al. 2006; Forsgren et al. 2013). In the last decade, PCR-based methods have been developed for the identification of bacterial culture or for the detection of the pathogen from hive specimens, i.e., hive debris, honeybee workers, and diseased brood (Govan et al. 1999; Dobbelaere et al. 2001; Alippi et al. 2002; Piccini et al. 2002; Ryba et al. 2009; Alippi et al.^ 2004^; De Graaf et al. 2006; Lauro et al. 2003; Han et al. 2008; Martínez et al. 2010; Chagas et al. 2010; Rossi et al. 2018; Biova et al. 2021). As mentioned above, most of the developed PCR methods have been used for taxonomical identification of both bacterial pathogens from the culture. Currently, the matrix-assisted laser desorption/ionization time-of-flight mass spectrometry (MALDI-TOF MS) widely used for microbial identification in diagnostic laboratories allows precise identification of both pathogens (Kopcakova et al. 2022). That technique provides cheap and rapid results (within minutes). Therefore, PCR-based identification of *P. larvae* from culture is not crucial. On the other hand, surveillance of both diseases requires detection of the pathogen in surrounding apiaries. In particular, in the initial phase of the disease, clinical symptoms may be difficult to detect, and physical examination of all beehives is time-consuming. Therefore, PCR-based technique brings an efficient tool for screening pathogens. In positive beehives, the brood can be examined in detail to confirm the disease.

Our newly developed assay is based on the detection of an insertion sequence (IS256) specific for *P. larvae* and a specific sequence of plasmid pMP1 of *M. plutonius*. Confirmation of *M. plutonius* and virulence determination can be performed by a simplex PCR detecting ETX/MTX2 family pore-forming toxin. Insertion sequences (ISs) are one of the bacterial mobile genetic elements. They are usually 700–2400 bp long consisting of invert repetitions and genes encoding the protein responsible for mobility of this element (transposase). Insertion sequences can be responsible for the inactivation of a gene due to its transposition into the promoter or open reading frame. IS can even contain transcriptional and translational termination signal and can block the expression of other genes. Genomes of some bacterial species can be rich with the presence of IS, containing even more than 10% of IS. Detection of bacterial agents due to IS is commonly used for diagnostics of *Mycobacterium tuberculosis*, increasing the sensitivity of the method (Niyaz Ahmed et al. 1998). Therefore, we selected a specific IS256 found in the genome of *P. larvae* in multiple copies to increase the sensitivity of the method especially for surveillance purposes.

Until now, most of the PCR assays have been targeted on a variable region of the 16S rRNA gene (Govan et al. 1999; Dobbelaere et al. 2001; Alippi et al. 2002; Piccini et al. 2002; Ryba et al. 2009) with low copy numbers in *P. larvae* genome. Using a BLAST search (http://www.ncbi.nlm.nih.gov/blast), a similarity of that variable 16S rRNA region between *P. larvae* and *Brevibacillus laterosporus* can be identified. Therefore, misidentification of *P. larvae* could be theoretically observed. To increase the reliability of classification, multigene analysis should be used (Berg et al. 2018). Similarly, sequencing of amplified 16S rRNA gene can be used (Rossi et al. 2018) but it further increases the cost of the assay and time to obtain final results.

Using a BLAST search, IS*256* gene is specific for *P. larvae* only; therefore, no further pathogen verification is needed. However, it should be mentioned that insertion sequences are, based on the definition, mobile genetic elements that can be spread horizontally in bacterial populations, usually via plasmids (Partridge et al. 2018). Currently, no horizontal gene transfer was demonstrated in *P. larvae* apart from bacteriophages (Tsourkas 2020). Therefore, for disease confirmation, it is necessary to identify clinical symptoms in a suspected bee colony. That condition, however, is requested for all laboratory diagnostic methods as the disease cannot be confirmed by laboratory diagnostics only.

For *M. plutonius*, we selected a plasmid-specific sequence that has been found in that bacterium only. Like the target used for the detection of *P. larvae*, plasmids can be spread horizontally as conjugative plasmids or mobilizable elements. Even though the co-infection of *P. larvae* and *M. plutonius* has never been described, the multiplex format of the reaction allows cheap surveillance in apiaries in a large geographical area for non-targeted detection of both diseases. Similarly, it can be also used for the detection of positive apiaries in previous contact with the disease. For final confirmation of American and/or European foulbrood, however, it is necessary to confirm positive PCR results with observation of clinical symptoms in a suspected apiary.

As an internal control, we selected a gene encoding for major royal jelly protein 1 as this gene is present in *A. mellifera*. That allows quality control of the whole diagnostic process including specimen collection, DNA isolation, and PCR. Simultaneous detection of honeybee-specific protein is important for countries where active surveillance of American and European foulbrood requires the elimination of diseased colonies as well as observation in specific areas around the apiary with a confirmed disease.

## Conclusion

A novel PCR assay for the detection of *P. larvae* and *M. plutonius* was developed and validated on specimens of hive debris, honeybee workers, and diseased broods. For *P. larvae*, we proposed insertion sequence IS*256* as a target gene and pMP1 plasmid sequence for *M. plutonius*. The IS256 occurs in the *P. larvae* genome in multiple copies allowing sufficient sensitivity (≈10^2^ CFU). That method can be used for screening of *P. larvae* and *M. plutonius* in honeybee colonies and for confirmation of American and European foulbrood in suspected colonies showing typical symptoms. PCR confirmation can reduce time-to-results from a week usually required for cultivation to 1 day allowing efficient control of those diseases.
